# Adaptation of the Clinical Global Impression for Use in Correctional Settings: The CGI-C

**DOI:** 10.3389/fpsyt.2019.00687

**Published:** 2019-09-18

**Authors:** Roland M Jones, Kiran Patel, Mario Moscovici, Robert McMaster, Graham Glancy, Alexander I.F. Simpson

**Affiliations:** ^1^Centre for Addiction and Mental Health (CAMH), Toronto, ON, Canada; ^2^Department of Psychiatry, University of Toronto, Toronto, ON, Canada

**Keywords:** correctional psychiatry, prison, mental health, assessment, rating scale, severity

## Abstract

**Background:** Provision of mental health care in correctional settings presents unique challenges. There is a need for a simple-to-use tool to measure severity of mental illness in correctional settings that can be used by mental health staff from different disciplines. We adapted the severity scale of the Clinical Global Impression for use in correctional settings, which we have called CGI-C, and carried out a reliability study.

**Method:** Clinical descriptions of typical inmate presentations were developed to benchmark each of the seven possible ratings of the CGI. Twenty-one case vignettes were then developed for study of inter-rater reliability, which were then rated using the CGI-C by five forensic psychiatrists (on three occasions) and 11 multidisciplinary health care clinicians (twice). The tool was introduced into clinical practice, and the first 57 joint assessments carried out by both a psychiatrist and a clinician in which a CGI-C was rated were compared to measure inter-rater reliability.

**Results:** We found very good inter-rater and test–retest reliability in all analyses. Gwet’s AC, calculated on initial ratings of the vignettes by the psychiatrists, was 0.85 (95% CI 0.81–0.90, *p* < 0.001) and 0.87 (95% CI 0.83–0.91, *p* < 0.001) for clinician ratings. Inter-rater reliability based on 57 joint face-to-face assessments of inmates showed Gwet’s AC coefficient of 0.93 (95% CI 0.88–0.97).

**Conclusion:** The CGI-C is simple to use, can be used by members of the multidisciplinary team, and shows high reliability. The advantage in correctional settings is that it can be used even with the most severely ill and behaviorally disturbed, based on observation and collateral information.

## Background

Mental disorder is common among people detained in correctional^1^ settings (1). Although numerous studies have reported on the prevalence of mental illness in correctional settings, very few have measured the severity (2–4). A valid scale to rate the severity of mental disorder serves three purposes: 1) to enable a clinician to concisely communicate cross-sectional clinical information to those providing care within and outside of the team; 2) to enable the treating clinicians to monitor clinical progress by serial measurement (this is particularly important when there are different clinicians involved in a case, for example, where there may be temporary or locum appointments due to difficulty in recruitment); and 3) for administrative and service planning purposes within an organization, for example, tracking the prevalence of inmates with a given level of severity, and to compare with other institutions and over time.

Several scales for rating the severity of psychopathology exist, such as the 24-item Brief Psychiatric Rating Scale (BPRS) (5), which can be administered by semi-structured interview, and the Positive and Negative Syndrome Scale (PANSS) (6), a 30-item rating scale for schizophrenia. The Jail Screening and Assessment tool (JSAT) (7), which is a widely used screening and triage structured professional judgement tool for use in corrections, incorporates 10 items modified from the BPRS. Current rating scales for psychopathology therefore require a fairly detailed mental state examination, which is often not possible to carry out in correctional settings, particularly remand settings due to the very high level of behavioral disorganization of the individuals, resulting in diminished ability to participate in a structured assessment.

The Clinical Global Impression Scale (CGI) (8) is one of the most widely used brief rating scales in mental health and pharmaceutical trials. The brevity and simplicity of the tool suggest that it may have utility for routine use in correctional settings. The CGI consists of three domains; Global Severity, Global Improvement, and Therapeutic Index. The Global Severity domain of the CGI is a single overall rating of severity of illness, which is rated on a seven-point scale rated from “No Mental Disorder”, to “Among the most severely ill patients”. There are also two global rating scales for Global Improvement (clinician’s impression of change) and Therapeutic Index (clinician’s impression of efficacy of treatment). The first reported study that measured the reliability of the CGI was by Dahlke et al. (9). Several studies have demonstrated the validity of the CGI by linkage to other rating scales such as the PANSS (10–12), the BPRS (5, 10), and the WHODAS (13). It has also been validated in video form compared with face-to-face scoring (14), and has been used to predict suicidal behavior (15).

The original CGI has however been criticized for having inadequate scale construction and item labels (16). In addition, it has been shown that clinicians use different parameters to judge the severity of mental disorder between patients in different settings. For example, Ortiz and colleagues (17) found that CGI ratings of severity for equivalent PANSS scores differed between ratings of inpatients and outpatients, possibly because the clinicians were using a different frame of reference for severity when judging global impression of patients in these different settings.

Given some of the limitations of the original CGI, modifications of the CGI have been made for assessment of patients with different conditions, including bipolar affective disorder (CGI-BP) (18), schizophrenia (CGI-SC) (19, 20), autism (OSU Autism CGI) (21), borderline personality disorder (CGI-BPD) (22), and depression (iCGI) (23).

The CGI has been used in correctional settings (24–26); however, to our knowledge, there have been no validation studies of the CGI with this population. The assessment and treatment of inmates in custodial settings is not directly comparable to work in hospital or outpatient settings. First, there are higher rates of morbidity (1). Second, the environment itself produces unique challenges (27). The patients who are most behaviorally disturbed and therefore most in need of mental health care are generally locked in their cells, sometimes necessitating psychiatric assessments being carried out through a window in a closed cell door, or through an open rectangular hatch in the inmate’s cell door (designed for passing food trays in and out). Assessment of the most severely ill patients, therefore, is often based on what is observed (the behavior of the inmate, the condition of the cell, and the reports of the correctional officers who have been observing them) at least as much as what is said by the inmate.

There is therefore a need for a brief tool that can be used to rate even the most severely unwell patients in custodial settings. This tool must be reliable and be able to be used by multidisciplinary staff and in research contexts. Our aim was to adapt and assess the reliability of the Global Severity scale of the CGI for use in correctional settings, which we have named the Clinical Global Impression—Corrections, abbreviated to CGI-C.

## Methods

### Study Setting

The Forensic Early Intervention Service (FEIS) is a team of 6 psychiatrists and 12 clinicians (comprising 3 nurses, 6 social workers, and 3 occupational therapists) in two provincial jails in Toronto, Canada, and provides assessment and triage of inmates who have or are suspected of having serious mental health needs, and case management for those patients where there are concerns pertaining to their fitness to stand trial or if they may be pursuing a defense of “not criminally responsible” under the Canadian Criminal Code. Every prisoner is screened at reception into custody using the Brief Jail Mental Health Screen (28) by correctional primary health staff, and those screening positive are referred to the FEIS service for further triage and assessment using the JSAT (7). Those that are determined to need either further assessment or meet the inclusion criteria of the FEIS service are referred to a FEIS psychiatrist for further assessment. If, on further assessment, the patient is determined to meet the criteria of the FEIS service, they are allocated a caseworker and a psychiatrist. The caseworker and psychiatrist who then follow the patient are typically those who carried out the initial assessments. FEIS provides service for remand inmates in one provincial jail for men (capacity of 1650) and one provincial jail for women (capacity of 300).

Research Ethical approval for use of routinely collected data for FEIS research was granted by the Centre for Addictions and Mental Health Research Ethics Board (# 035/2018-01). Consent was not sought directly from participants; no identifiable information was retained or is presented in this manuscript.

### Study Design

First, two of the authors (RJ and MM) developed clinical descriptions of typical inmate presentations spanning the range of severity to correspond to the seven possible ratings of the CGI, ranging from “No mental disorder” to “Among the most severely ill patients”. These clinical descriptions were then revised and agreed by consensus among five experienced forensic psychiatrists who work in correctional settings. It was decided to allow for both collateral information (such as is often provided by corrections officers who have observed the patient) and information that is gathered by the assessor, by direct observation or by interview, to be incorporated into the ratings. A brief user’s guide was developed for instructions on rating and was revised several times by consensus (29).

Second, the five forensic psychiatrists who work in correctional settings provided brief anonymized composite clinical vignettes of patients typically seen within a correctional setting. The lead author reviewed and adapted the vignettes to ensure there was a full range in severity of clinical presentations, that a variety of diagnoses were represented, and that there was a balance of gender. In total, 21 clinical vignettes were selected for study of inter-rater reliability of the CGI-C, which included vignettes that described individuals with psychosis, depression, drug withdrawal, anxiety, obsessive–compulsive disorder, and cognitive impairment.

The forensic psychiatrists were then asked to rate each of the clinical vignettes using the CGI-C. The vignettes were loaded into an electronic survey program (30) and were presented to each assessor in a random order as generated by the program. Participants recorded their rating, the results of which were electronically stored and made available to the lead author. The user guide was revised based on the feedback and results from the survey.

Approximately 3 months later, the psychiatrists were asked to rate the same vignettes without having access to their previous ratings (again presented in random order). We measured interrater reliability of these ratings and measured test–retest reliability. We made minor modifications to the user guide and to the item descriptions of the scale following these ratings.

We then provided a 1-h training session on the CGI-C to members of the multidisciplinary team of clinicians who work in the FEIS service. After training, the clinicians were asked to each rate the 21 clinical vignettes, in a format identical to that used for gathering the ratings of the psychiatrists. We measured the inter-rater reliability of these ratings, following which, a 1-h feedback session was provided to review the ratings of the vignettes.

We then implemented the CGI-C in the clinical setting for the first 60 joint assessments in which both a psychiatrist and clinician assessed a patient simultaneously and rated independently of each other, and we compared their CGI-C ratings.

Based on further discussion, we decided to make a minor revision to the wording of part of the user guide. To test whether this change affected the reliability of the rating, we requested all participants to again rate the vignettes with reference to the new version of the user guide, and we calculated the inter-rater and test–retest reliability of these ratings. The development of the tool and measurement of reliability took place between February 2018 and January 2019.

### Statistical Analysis

We calculated inter-rater reliability using Gwet’s AC (31). Gwet’s AC is considered to be an improvement on Cohen’s Kappa due to improved correction for chance agreement and is more robust when there is less variation in ratings between raters (32, 33). The seven-point CGI-C scale is ordinal, and therefore ordinal weighted coefficients were calculated using *kappaetc* command in Stata (version 14) (34). Interpretation of coefficient values as described by Altman (35) is as follows: < 0.2 = poor, 0.2–0.4 = fair, 0.4–0.6 = moderate, 0.6–0.8 = good, and 0.8–1.0 = very good. As well as reporting the coefficient, we categorized the coefficient using the probabilistic categorization of coefficient that takes into account the variance of the estimate, as described by Gwet (31).

## Results

The inter-rater reliability coefficient, Gwet’s AC, calculated on the first set of ratings carried out by the forensic psychiatrists was 0.85 (95% CI 0.81–0.90, *p* < 0.001). The probability that the inter-rater reliability coefficient falls in the “Very Good” category was greater than 0.99. The inter-rater reliability coefficient, Gwet’s AC, calculated on the second set of ratings by the forensic psychiatrists was 0.89 (95% CI 0.85–0.94, *p* < 0.001). The probability that the inter-rater reliability coefficient falls in the “Very Good” category was again greater than 0.99. The intra-rater reliability (test–retest reliability rating) for each of the five psychiatrists comparing their first and second ratings was also very good, and the range of coefficients was between 0.86 and 0.91.

The inter-rater reliability was then calculated based on the ratings of the 21 vignettes by the 11 clinicians. The Gwet’s AC was 0.87 (95% CI 0.83–0.91, *p* < 0.001). The probability that the ratings fell in the “Very Good” category was >0.99.

We then calculated the inter-rater reliability of ratings during joint face-to-face assessments of inmates (see **Table 1**). There were 60 joint patient assessments carried out by 12 clinicians and six psychiatrists. Each clinician jointly assessed 5 cases with one of the psychiatrists. In three cases, only one rater recorded their rating, leaving 57 unique cases that were rated by both a psychiatrist and clinician. One psychiatrist rated 25 of the cases and another rated 13. The four remaining psychiatrists carried out 8, 7, 5, and 2 assessments, respectively. The median score rated by the psychiatrists was 4 (range 2–7). All of the ratings carried out by clinicians numbered 2, 8, and 9 in **Table 1** were conducted on female patients, the remainder on males.

**Table 1:** Inter-rater reliability ratings of patients between clinician and psychiatrist, by clinician.

Clinician	Number of cases rated	Gwet’s AC coefficient	95% CI
1	5	0.95	0.86–1.0
2	5	0.86	0.64–1.0
3	4	1.0	1.0–1.0
4	5	1.0	0.31–1.0
5	4	0.92	0.75–1.0
6	5	0.98	0.99–1.0
7	5	0.95	0.83–1.0
8	5	0.94	0.83–1.0
9	5	0.97	0.91–1.0
10	5	0.97	0.88–1.0
11	4	0.87	0.52–1.0
12	5	0.93	0.81–1.0
Total	57	0.93	0.88–0.97

The inter-rater reliability of the patient assessments using Gwet’s AC coefficient was 0.93 (95% CI 0.88–0.97). The probability of being in the “Very Good” category was >0.99. The AC coefficients for each of the clinicians are shown in **Table 1**, and those for the psychiatrists are shown in **Table 2**.

**Table 2:** Inter-rater reliability ratings of patients between clinician and psychiatrist, by psychiatrist.

Psychiatrist	Number of cases rated	Gwet’s AC coefficient	95% CI
1	22	0.95	0.91–0.98
2	7	0.93	0.79–1.0
3	8	0.95	0.87–1.0
4	13	0.94	0.75–1.0
5	2	0.89	0.89–0.89
6	5	0.91	0.67–1.0
Total	57	0.93	0.88–0.97

Finally, following a slight modification to the wording of part of the user guide, we requested that psychiatrists and clinicians re-rated the vignettes using the updated guide. We calculated the inter-rater reliability of the 21 clinical vignettes and test–retest reliability using the final version of the guide. Four psychiatrists and 13 clinicians rated the vignettes. Gwet’s AC coefficient was 0.89 (95% CI 0.85–0.92). With regard to test–retest reliability, psychiatrists had rated the vignettes three times and so we compared the first and third scores rated, whereas clinicians had rated the vignettes twice, and so we compared the first and second ratings. In all cases, there was very good test–retest reliability (range = 0.85–0.90).

## Discussion

This paper describes our adaptation of the CGI severity rating scale for use in correctional settings, the CGI-C. We developed a user guide and benchmarked each scale item using clinical descriptions based on the range and type of cases encountered in correctional settings. We revised and refined the guide and tested the inter-rater reliability of ratings on clinical vignettes and during routine clinical practice. We found that this tool has very good inter-rater and test–retest reliability. We believe that there is a need for such a tool that can be quickly and easily administered routinely in correctional settings. One of the most important features of this tool is that it can be used to rate those who are most severely ill and who are otherwise unable to cooperate in a clinical assessment due to their severe psychopathology. We found that it was quick and easy to use, was equally reliable when used to rate male and female patients, and could be rated equally well by different members of the multidisciplinary team.

We believe that this tool fills a significant gap in both routine correctional mental health practice and research on mental illness in correctional settings, where there is no reported use of severity measures in routine practice or as an accepted research tool. Rarely have large-scale epidemiology studies included such a measure in their designs. The Global Assessment of Functioning Scale (GAF) has been subject to criticism regarding its reliability and validity and has been dropped from DSM 5, and there have been no previous validation studies on the CGI in correctional settings. The routine use of a rapid measure of severity may be of great value in meeting the abovementioned purposes of severity and progress measurement in routine practice, and appears feasible for service planning and research.

## Limitations and Recommendations

The CGI-C is less informative than more detailed measures of psychopathology, which should also be used where indicated, and where the clinical presentation and logistical considerations allow. The CGI-C does not replace more detailed tools but is sufficiently quick and easy to rate that it could be done routinely on all cases.

We have not tested the validity of the CGI-C by comparing it against other measures. The validity of the original CGI has however been measured extensively, and it would be expected that the addition of our item descriptors would not diminish the validity of the tool; however, we recommend that further research is needed to assess the validity of the CGI-C in this population. In addition, we have not measured the validity as compared with real clinical outcomes, such as need for admission to hospital.

The original CGI has three domains, severity, global improvement, and therapeutic efficacy. We decided *a priori* to adapt only the first domain, global severity, for use in corrections. The improvement scale has been criticized for its psychometric properties, and in our view, having both a rating scale for severity and a separate one for improvement has dubious validity. An objective rating of severity that is sensitive to change is likely to be far more useful and have greater validity and reliability than a global impression of change, particularly when there are multiple raters and multiple episodes of care for a given client as often is the case in correctional settings. In addition, the therapeutic improvement scale is not considered to be useful in the correctional setting on a routine basis, though could conceivably be used if required to assess the impression of efficacy of a given course of treatment. Our work in assessing the inter-rater reliability of the CGI-C on patients has been carried out cross-sectionally. Although we believe that it is likely to be sensitive to change, further work is recommended to investigate sensitivity to change in correctional settings.

Finally, although we carried out this study in two jails, we recommend that further study of the utility and validity of the CGI-C be carried out in other correctional institutions to ensure that the results are generalizable.

## Conclusion

Our adaptation of the CGI severity scale for use in correctional settings, the CGI-C, is quick and simple to use, can be used by members of the multidisciplinary team, and shows high inter-rater and test–retest reliability. The advantage in correctional settings is that it can be used routinely, even with the most severely ill and behaviorally disturbed inmates, based on observation and collateral information. It may well fill an important gap in correctional mental health care, service planning, and research.

## Data Availability Statement

The data that support the findings of this study are available from the corresponding author upon reasonable request.

## Ethics Statement

The studies involving human participants were reviewed and approved by Centre for Addiction and Mental Health Research Ethics Board, Toronto, Ontario. Written informed consent for participation was not required for this study in accordance with the national legislation and the institutional requirements.

## Author Contributions

RJ designed the study, carried out data collection, data analysis, and preparation of the first and revised drafts of the manuscript. KP contributed to the study design, carried out data collection, and contributed to the preparation and editing of the manuscript. MM contributed to the study design, carried out data analysis, and contributed to the preparation and editing of the manuscript. RM contributed to the data collection and contributed to the preparation and editing of the manuscript. GG contributed to the data collection and contributed to the preparation and editing of the manuscript. AS contributed to the design of the study, carried out data collection, and contributed to the preparation and editing of the manuscript. All authors have read and have approved the manuscript.

## Funding

RJ’s work was supported in part by an Academic Scholar Award from the University of Toronto.

## Conflict of Interest

The authors declare that the research was conducted in the absence of any commercial or financial relationships that could be construed as a potential conflict of interest.

## Footnotes

1 In North America, prisons and jails are known as correctional institutions, and hereafter the term corrections will be used to mean prisons, jails, detention centers, and other forms of criminal justice detention institutions.
